# Palliative care consultations in head and neck cancer

**DOI:** 10.1038/sj.bjc.6604086

**Published:** 2008-01-22

**Authors:** S Stewart

**Affiliations:** 1 Imperial College of London, London, UK

Palliative Care Consultations in head and neck cancer is the fifth book in a paperback series aimed at educating health-care professionals about different aspects of palliative care. The previous books cover haemato-oncology, gynaecological oncology, brain tumours and breast cancer.

This book, like others in the series, comprises a collection of topics written by different authors. Each chapter is written as a separate article and there is little to link one chapter with any other. The chapters are however easy to read, informative, and well referenced. There is an excellent review on the non-surgical management of advanced head and neck cancer and valuable chapters concerning the management of airways, improving swallowing and communication, and providing nutritional support.

Although this book is aimed at palliative care, many of the chapters offer valuable information applicable to patients undergoing active surgical or oncology treatment. I found this book a valuable source of information and would recommend it to any health-care professional engaged in managing head and neck cancer patients.

